# Rapid and accurate detection of SARS-CoV-2 using the RHAM technology

**DOI:** 10.1038/s41598-023-49733-7

**Published:** 2023-12-20

**Authors:** Zhuo Xiao, Xiaoli Liu, Xiaolong Kang, Yaoheng Feng, Lijun Zheng, Chong Chen

**Affiliations:** Guangzhou Pluslife Biotech Co., Ltd., No. 6 Lianhuayan Road, Huangpu District, Guangzhou, 510700 Guangdong China

**Keywords:** Assay systems, Diagnosis

## Abstract

Rapid and sensitive detection of pathogens is of utmost importance in interrupting the transmission chain of infectious diseases. In recent years, this has proven to be vital during the coronavirus disease (COVID-19) global pandemic that put countless lives at risk. Numerous molecular diagnostic methods were used, including RT-PCR, NASBA, E-SDA, E-RCA, LAMP, and RPA. However, these technologies potentially require primer optimization and complex instruments. Here, we propose the RHAM (RNase Hybridization-Assisted amplification) system as a rapid, specific, and sensitive molecular diagnosis platform. Combining the LAMP and RNase HII-mediated fluorescent reporter, the RHAM system can amplify and visualize the target in one isothermal system with high sensitivity (5 × 10^2^ copies/mL). There was no cross-reactivity with other common respiratory viruses. Analysis of clinical samples revealed the RHAM system to generate positive signals within 15 min without false positive or negative results. The present study shows that RHAM is not only an ideal platform for detecting pathogens, such as SARS-CoV-2 but can be potentially applied in POCT settings.

## Introduction

Infectious diseases continue to be a long-term threat and burden to global public health. During the twentieth century, the influenza virus triggered several epidemics, causing millions of deaths^1,2^. In the twenty-first century, the prevalence of novel coronavirus led to economic recessions in 2020–2022. By May 2023, more than seven million individuals were infected. Other than acute respiratory symptoms and potential health-related effects, infectious viruses also induce chronic genital tract and nasal cavity infections, which may cause cervical and nasopharyngeal carcinoma, leading to greater health consequences to the patient compared to the respiratory pathogen alone^3^.

Currently, PCR, a molecular biologic technique, remains the gold standard for the identification of many viral pathogens and is recommended by clinical guidelines given its high sensitivity and specificity^4–8^. Due to the requirement of well-trained technicians, expensive equipment, and a specific laboratory environment, PCR testing can be somewhat less accessible and its application is limited in most developing countries, as well as in clinical and primary healthcare settings in developed countries without qualified technicians^9^. In contrast to PCR, which requires multiple thermal cycles, isothermal amplification can be performed under a constant temperature without complicated procedures^9–11^. Further, equipment costs for isothermal amplification are much lower than that of PCR. Isothermal nucleic acid amplification technology provides a rapid and simple experimental approach similar to the rapid antigen test (RAT); however, its sensitivity and specificity are far superior, making it a promising technology to develop low-cost diagnostic tools^12,13^.

Notably, RNase HII combined with blocked primers containing a single ribonucleotide residue which are activated via cleavage by the enzyme, can increase the sensitivity and specificity of the detection of SNPS in rhPCR systems and prevent primer-template independent amplification^14^. Based on these properties, we developed a new isothermal amplification method termed RHAM (RNase HII-assisted Amplification), which consists of Loop-Mediated Isothermal Amplification (LAMP)-mediated exponential amplification with an RNase HII reporter for signal visualization in a single reaction. In the initial reaction, with the assistance of the conventional LAMP primer set, the target sequence is exponentially amplified by Bst DNA polymerase^15^. In the latter stage, the ribonucleotide-containing fluorescent probe, labelled with a fluorophore and quencher, then hybridizes with the amplification product, in which the 5′ to the ribonucleotide can be recognized and nicked by RNase HII within the context of DNA–probe duplex. The digested fluorescent probe then dissociates from the targeted amplicon, resulting in the release of the fluorescent group from the quenching group, leading to an increase in the fluorescence signal, which can be read and recorded by a qPCR machine and a positive test result obtained within 10 min.

This novel method can be used for the rapid, sensitive, and specific detection of pathogens, such as severe acute respiratory syndrome coronavirus type 2 (SARS-CoV-2). The present study demonstrates that RHAM can detect the extracted nucleic acid rapidly, sensitively, and specifically, and also directly detect clinical samples without nucleic acid extraction. RHAM provides reliable technical support for the development of a sensitive, rapid, convenient, and portable POCT product and provides a broad prospect for the on-site detection of pathogens.

## Results

### Development of the RHAM system for detecting SARS-CoV-2 RNA

**Figure 1:**
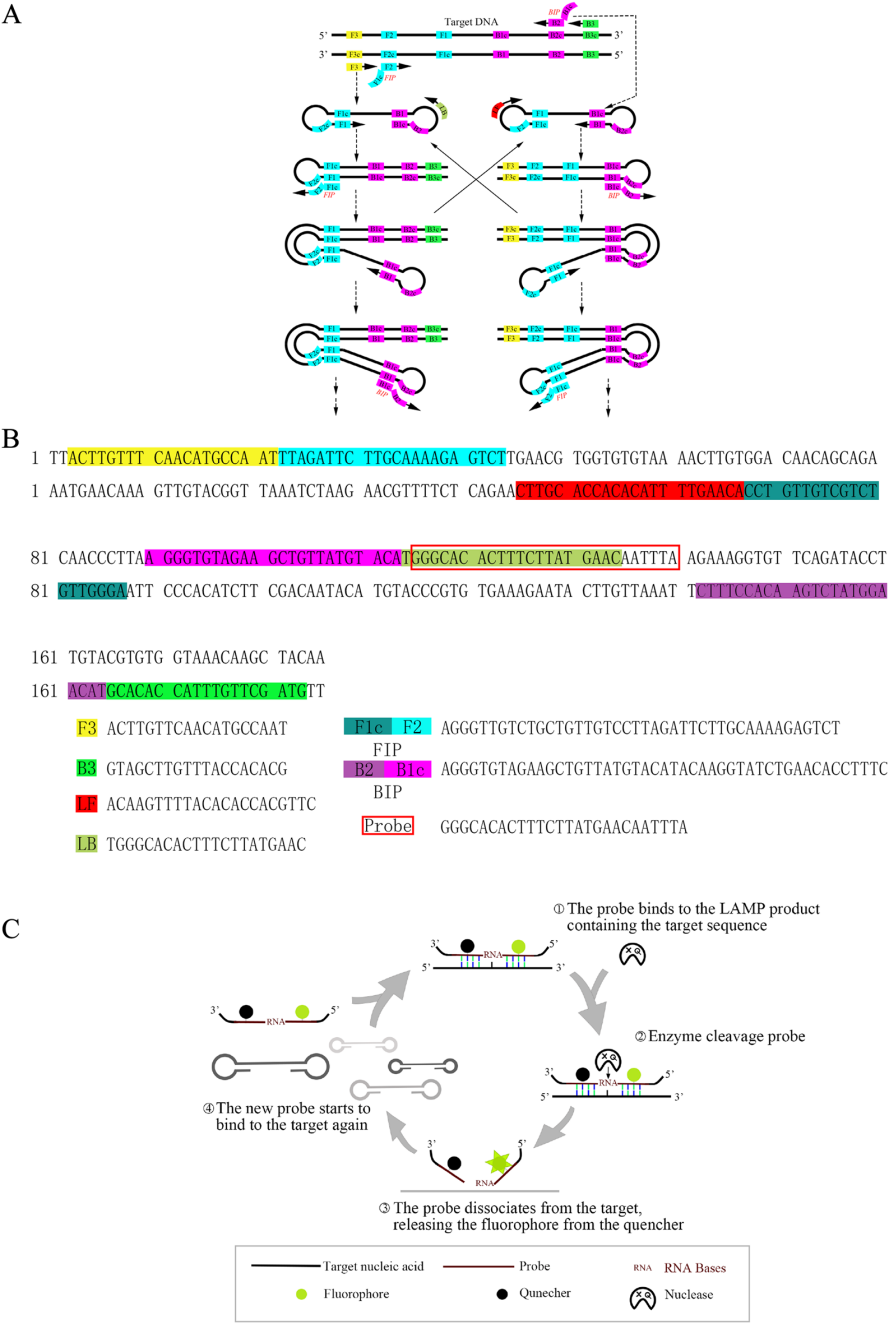
Schematic overview of the RHAM technology. (**A**) Schematic overview of the fluorescence-based LAMP technology. (**B**) Schematic diagram showing the targeted sequence and primer designed against Orf1ab. (**C**) Principle of RHAM. In RHAM, LAMP is coupled with the RNase HII reporting system for signal readout in a single tube. Aided by a LAMP primer set comprising six primers, the target sequence is reverse transcribed and exponentially amplified by Bst DNA polymerase. During the exponential amplification, because of the strand displacement ability of Bst DNA polymerase, part of the amplification product turns into a single-stranded state. The ribonucleotide containing fluorescent probe, labeled with a fluorophore and quencher, can hybridize to the single-stranded amplification product and be cleaved by RNase HII, releasing the fluorophore from the quencher and emitting the fluorescent signal. The remaining single-stranded product can hybrid with the new probe and trigger a new cycle of signal amplification.

In the fluorescence-based LAMP detection protocol, reverse transcription of the target genome generates complementary DNA (cDNA). Meanwhile, in the single tube reaction, LAMP primers anneal to the cDNA and the cDNA is amplified using Bst DNA polymerase. The resulting product contained is a repeat of the amplicon linked by a single-stranded loop in a long concatemer (Fig. 1A,B)^16^. To enhance LAMP detection, RNase HII and the fluorescent probe were added to the LAMP reaction mix for the specific detection of amplicons targeting Orf1ab and N gene. During the LAMP process, the ribonucleotide-containing fluorescent probe, labeled with a fluorophore and a quencher, can hybridize with the single-stranded LAMP products. Upon recognition by RNase HII, the RNA–DNA hybrid was then cleaved, resulting in the release of the fluorophore (Fig. 1C). We combined the RNase HII, ribonucleotide-containing fluorescent probe with the LAMP system (hereafter named RHAM).

**Figure 2:**
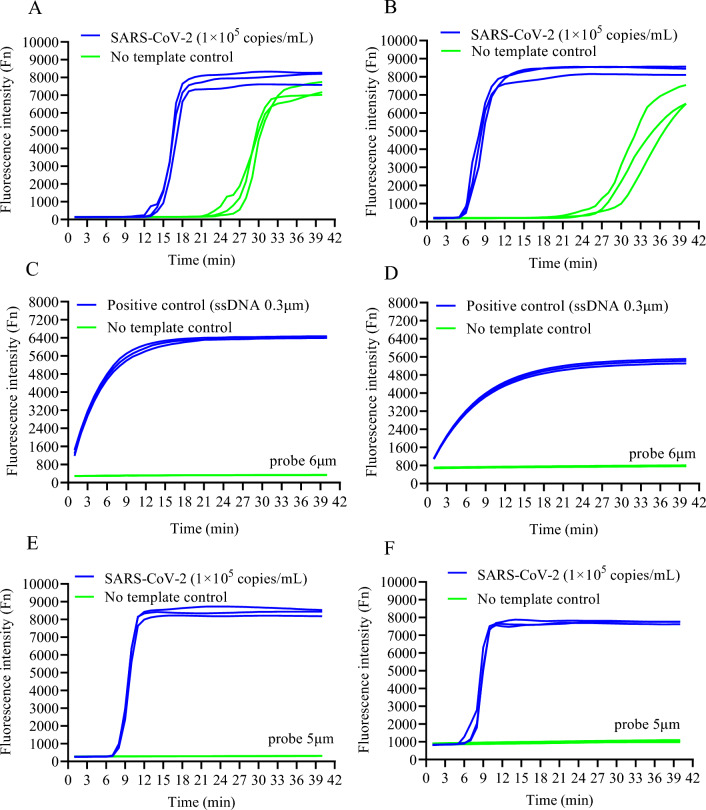
Development of the RHAM system for robust SARS-CoV-2 detection. (**A**) Example of fluorescence-based LAMP targeting Orf1ab with SARS-CoV-2 RNA (blue) and no template control (green). (**B**) Example of fluorescence-based LAMP targeting N gene with SARS-CoV-2 RNA (blue) and no template control (green). (**C**) Detecting Orf1ab single-stranded DNA with RNase HII. A single-stranded DNA (ssDNA) complementary to the probe is synthesized to evaluate the cleavage ability of RNase HII. RNase HII and probe were incubated at 65 °C with (blue) and without (green) ssDNA. (**D**) Detecting N gene single-stranded DNA with RNase HII. A single-stranded DNA (ssDNA) complementary to the probe is synthesized to evaluate the cleavage ability of RNase HII. RNase HII and probe were incubated at 65 °C with (blue) and without (green) ssDNA. (**E**) Combination of LAMP and RNase HII reporting system. RNase HII and the probe targeting Orf1ab were added to the LAMP reaction mixture. With SARS-CoV-2 RNA (blue); no template control (green). (**F**) Combination of LAMP and RNase HII reporting system. RNase HII and the probe targeting N gene were added to the LAMP reaction mixture. With SARS-CoV-2 RNA (blue); no template control (green).

To compare LAMP and RHAM, experiments were performed for the detection of SARS-CoV-2. The real-time kinetics of positive (purified SARS-CoV-2 RNA reference, 500 copies/reaction) and negative samples (no template control) targeting the Orf1ab gene or N gene in LAMP reactions were monitored. Positive samples were found to display fluorescence after incubation for 15 min; however, both positive and negative samples showed similar fluorescence intensities after incubation for 27 min due to amplification of primer dimer (Supplementary Fig. 1), indicating the risk of a high false-positive rate, making it difficult to discriminate negative and low concentration positive result (Fig. 2A,B). To evaluate the performance of RNase HII, a positive fluorescent signal can be observed when adding both the ribonucleotide-containing fluorescent probe and the target single-stranded (ssDNA) compared to the negative control (without ssDNA; Fig. 2C,D). Next, the RHAM system results showed that the single pot system can robustly detect purified SARS-CoV-2 RNA targeting either Orf1ab or N gene in less than 10 min without a false positive signal in comparison to the LAMP system (Fig. 2E,F). Therefore, the RHAM system showed an evident improvement compared to LAMP. The above data indicate that time-to-positivity (TTP) value of RHAM is superior to or equal to LAMP and has stronger specificity compared with LAMP method for SARS-CoV-2 RNA detection. It is worth noting that the well-designed LAMP assays will not produce secondary structures and non-specific amplification products. We provide the RHAM assays as a choice for the primers which might generate non-specific amplification in the other assays, so that the available primers can be more easily selected.

### RHAM is highly sensitive and specific

**Figure 3:**
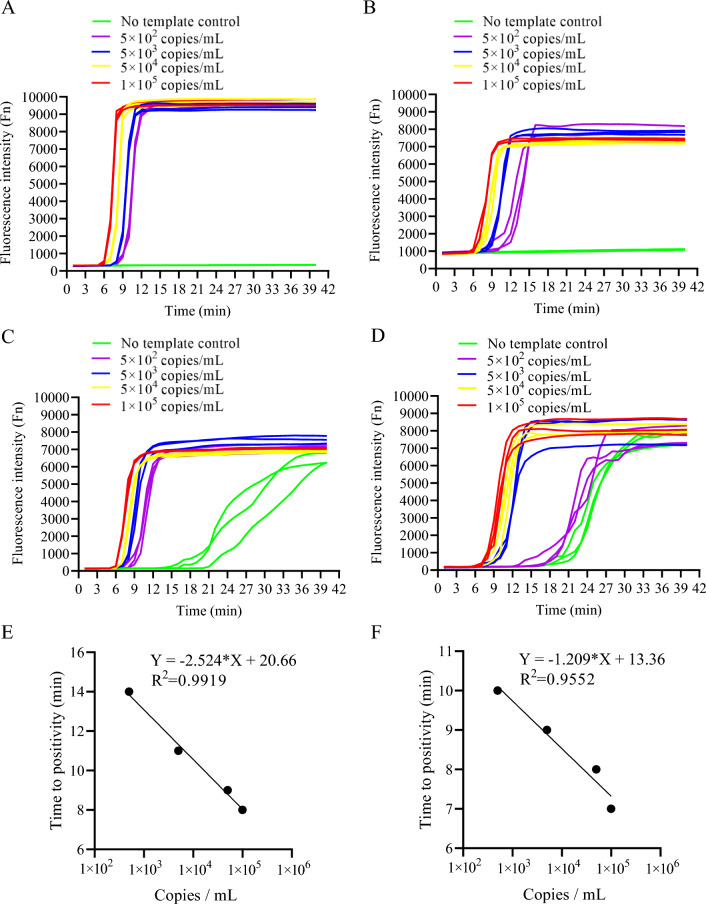
RHAM demonstrates high sensitivity. (**A**) The sensitivity of RHAM was evaluated by adding 10 μL of SARS-CoV-2 nucleic acid with different concentrations to the RHAM reaction mixture targeting Orf1ab (25 μL reaction volume). 5 × 10^5^ copies/mL (red), 5 × 10^4^ copies/mL (yellow), 5 × 10^3^ copies/mL (blue), 5 × 10^2^ copies/mL (purple), and no template control (NTC, green). (**B**) The sensitivity of RHAM was evaluated by adding 10 μL of SARS-CoV-2 nucleic acid with different concentrations to the RHAM reaction mixture targeting N gene (25 μL reaction volume). 1 × 10^5^ copies/mL (red), 5 × 10^4^ copies/mL (yellow), 5 × 10^3^ copies/mL (blue), 5 × 10^2^ copies/mL (purple), and no template control (NTC, green). (**C**) The sensitivity of LAMP was evaluated by adding 10 μL of SARS-CoV-2 nucleic acid with different concentrations to the LAMP reaction mixture targeting Orf1ab (25 μL reaction volume). 1 × 10^5^ copies/mL (red), 5 × 10^4^ copies/mL (yellow), 5 × 10^3^ copies/mL (blue), 5 × 10^2^ copies/mL (purple), and no template control (NTC, green). (**D**) The sensitivity of LAMP was evaluated by adding 10 μL of SARS-CoV-2 nucleic acid with different concentrations to the LAMP reaction mixture targeting N gene (25 μL reaction volume). 5 × 10^5^ copies/mL (red), 5 × 10^4^ copies/mL (yellow), 5 × 10^3^ copies/mL (blue), 5 × 10^2^ copies/mL (purple), and no template control (NTC, green). (E) the correlation between TTP and copy number of SARS-CoV-2 in RHAM amplification targeting Orf1ab. (**F**) the correlation between TTP and copy number of SARS-CoV-2 in RHAM amplification targeting N gene.

**Figure 4:**
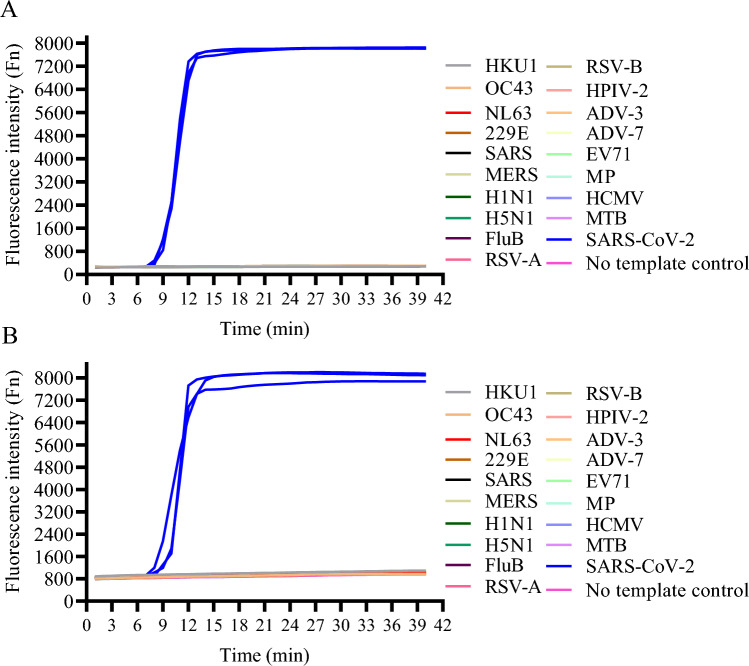
Evaluation of RHAM specificity. (**A**) The specificity of RHAM targeting Orf1ab was evaluated by testing the nucleic acids of other respiratory pathogens, (**B**) The specificity of RHAM targeting N gene was evaluated by testing the nucleic acids of other respiratory pathogens. All pathogens including coronavirus (HKU1, OC43, NL63, 229E), SARS coronavirus, MERS coronavirus, influenza A H1N1, H5N1, H7N9, and H9N2, influenza B virus, respiratory syncytial virus types A and B, human parainfluenza virus type II, adenovirus types 3 and 7, enterovirus EV71, *Mycoplasma pneumoniae*, Epstein-Barr virus, human cytomegalovirus, and *Mycobacterium tuberculosis*. 10 μL of nucleic acid was added to the RHAM reaction mixture (25 μL reaction volume).

RHAM was established successfully for the rapid detection of SARS-CoV-2; however, its performance required further clarification. To assess the sensitivity and specificity of RHAM, its detection limit was examined using SARS-CoV-2 RNA samples. A series of diluted SARS-CoV-2 RNA samples (1 × 10^5^–5 × 10^2^ copies/mL) were prepared, with each added to 25 μL of RHAM reaction mix targeting Orf1ab (Fig. 3A) or N gene (Fig. 3B). We then monitored the fluorescence of each dilution and calculated their coefficient of variations (CVs). All SARS-CoV-2 RNA positive samples showed fluorescence signals within 25 min. Both LAMP and RHAM amplified all the tested dilutions with similar TTP values. The no template control did not display any fluorescence signal in RHAM (Fig. 3A,B). However, using the same primer sets, LAMP may produce false positive signal in ~ 30 min (Fig. 3C,D). The TTP showed good correlation to the concentration of templates in the RHAM system (Fig. 3E,F). The specificity of the orf1ab and N gene primer sets and probe was also analyzed for SARS-CoV-2 diagnostics (Fig. 4A,B). Nucleic acids of other respiratory pathogens, including coronavirus (HKU1, OC43, NL63, 229E), SARS coronavirus, MERS coronavirus, influenza A H1N1, H5N1, H7N9 and H9N2, influenza B Virus, respiratory syncytial virus types A and B, human parainfluenza virus type II, adenovirus types 3 and 7, enterovirus EV71, *Mycoplasma pneumoniae*, Epstein-Barr virus, human cytomegalovirus, and *Mycobacterium tuberculosis* were synthesized. The extracted nucleic acids were added to the RHAM reaction mix. There was no cross-reactivity with other respiratory pathogens, with only SARS-CoV-2 displaying fluorescent signals at a run time of up to 40 min (Fig. 4A,B), thus reflecting the specificity of the RHAM system. These data prove that RHAM is a rapid, sensitive, and specific technology for nucleic acid detection.

### Fast and accurate detection of SARS-CoV-2 from clinical samples by RHAM without RNA extraction

**Figure 5:**
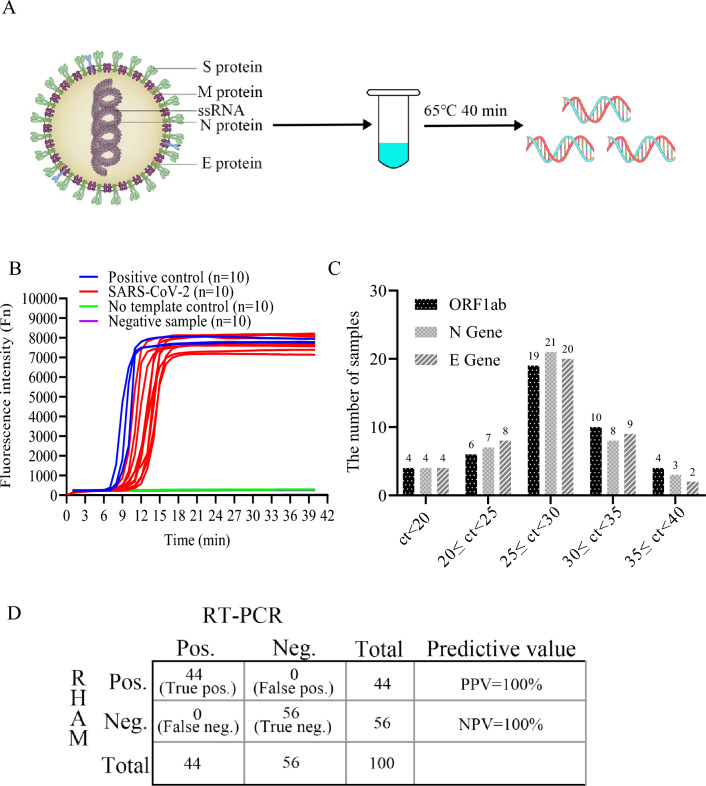
Performance of RHAM in the evaluation of clinical samples. (**A**) Schematic overview of RHAM detection of SARS-CoV-2 in clinical specimens. (**B**) Detection of SARS-CoV-2 in human clinical samples using RHAM. Ten SARS-CoV-2 positive and ten negative samples were analyzed. For RHAM detection, 5 μL of the medium was mixed with 5 μL lysis buffer. The lysis product was added to the reaction buffer, with a final reaction mixture of 25 μL. Positive control (blue); No template control (green); positive samples (red). (**C**) Ct value distribution of RT-qPCR corresponding to positive samples in 100 samples (Some of the genes in the same SARS-CoV-2 sample were NoCt or positive, resulting in a total of 43 positive samples for the same gene). (**D**) Positive and negative rates of RHAM based on RT-PCR.

To assess the ability of RHAM in POCT, the detection of clinical nasopharyngeal samples without RNA extraction was performed. Nasopharyngeal swabs were collected from patients and added to the storage medium. Positive and negative samples were first determined to use commercially available RT-PCR kits. To evaluate the performance of RHAM, 10 SARS-CoV-2 positive and 10 SARS-CoV-2 negative samples were included and randomized. For RHAM detection, 5 μL of the medium was mixed with 5 μL of lysis buffer. The mixture was then added to the RHAM reaction mix to a final total volume of 25 μL (Fig. 5A). Positive results were obtained for all RT-PCR confirmed positive samples using the RHAM platform within 25 min (Fig. 5B). Moreover, all the SARS-CoV-2 negative samples did not demonstrate any fluorescence signals at a run time of up to 40 min (Fig. 5B). And among the test of 100 clinical samples, RHAM showed 100% agreement with RT-PCR in both positive and negative samples (Fig. 5C,D and Table 1). The data demonstrate 100% accuracy in SARS-CoV-2 diagnosis using the RHAM platform, indicating that the method is a robust and accurate technology to detect SARS-CoV-2 in clinical applications.

**Table 1:** Clinical sample test information sheet referred to Fig. 5

	RHAM test	RT PCR-LR	ORF1ab (green)	N gene (orange)	E gene (yellow)	IC (red)	Conformance
1	Positive	Positive	21.95	21.14	21.82	26.03	Consistent
2	Negative	Negative	NoCt	NoCt	NoCt	28.56	Consistent
3	Negative	Negative	NoCt	NoCt	NoCt	31.50	Consistent
4	Positive	Positive	18.11	16.31	15.27	23.03	Consistent
5	Positive	Positive	29.06	26.14	24.24	25.89	Consistent
6	Positive	Positive	27.10	25.30	27.30	25.30	Consistent
7	Negative	Negative	NoCt	NoCt	NoCt	26.60	Consistent
8	Positive	Positive	16.99	15.59	14.34	23.97	Consistent
9	Positive	Positive	30.31	27.72	27.57	26.89	Consistent
10	Positive	Positive	27.29	25.03	25.26	26.77	Consistent
11	Positive	Positive	25.50	26.40	27.10	25.30	Consistent
12	Negative	Negative	NoCt	NoCt	NoCt	24.90	Consistent
13	Positive	Positive	26.70	24.50	27.10	23.50	Consistent
14	Positive	Positive	24.75	23.26	22.05	25.17	Consistent
15	Positive	Positive	25.70	27.40	24.60	25.30	Consistent
16	Negative	Negative	NoCt	NoCt	NoCt	25.30	Consistent
17	Negative	Negative	NoCt	NoCt	NoCt	26.40	Consistent
18	Negative	Negative	NoCt	NoCt	NoCt	31.50	Consistent
19	Positive	Positive	18.11	16.31	15.27	23.03	Consistent
20	Positive	Positive	29.06	26.14	24.24	25.89	Consistent
21	Positive	Positive	27.10	25.30	27.30	25.30	Consistent
22	Negative	Negative	NoCt	NoCt	NoCt	24.50	Consistent
23	Positive	Positive	16.99	15.59	14.34	23.97	Consistent
24	Positive	Positive	30.31	27.72	27.57	26.89	Consistent
25	Negative	Negative	NoCt	NoCt	NoCt	26.70	Consistent
26	Negative	Negative	NoCt	NoCt	NoCt	27.10	Consistent
27	Positive	Positive	26.40	26.50	26.20	24.50	Consistent
28	Positive	Positive	25.50	26.10	27.30	26.10	Consistent
29	Positive	Positive	24.30	24.80	24.60	24.20	Consistent
30	Positive	Positive	25.30	25.90	25.40	25.20	Consistent
31	Positive	Positive	36.00	34.00	32.00	32.00	Consistent
32	Positive	Positive	34.00	33.00	32.00	30.00	Consistent
33	Positive	Positive	39.32	44.80	31.65	39.49	Consistent
34	Positive	Positive	36.20	37.24	30.23	38.86	Consistent
35	Positive	Positive	24.80	23.64	28.12	25.42	Consistent
36	Positive	Positive	NoCt	38.88	35.91	32.41	Consistent
37	Positive	Positive	28.80	39.64	NoCt	36.91	Consistent
38	Negative	Negative	NoCt	NoCt	NoCt	25.05	Consistent
39	Negative	Negative	NoCt	NoCt	NoCt	29.10	Consistent
40	Negative	Negative	NoCt	NoCt	NoCt	29.13	Consistent
41	Negative	Negative	NoCt	NoCt	NoCt	29.01	Consistent
42	Negative	Negative	NoCt	NoCt	NoCt	26.06	Consistent
43	Negative	Negative	NoCt	NoCt	NoCt	30.08	Consistent
44	Negative	Negative	NoCt	NoCt	NoCt	28.03	Consistent
45	Negative	Negative	NoCt	NoCt	NoCt	25.18	Consistent
46	Positive	Positive	29.33	29.04	28.99	25.16	Consistent
47	Positive	Positive	34.25	34.01	33.99	24.02	Consistent
48	Negative	Negative	NoCt	NoCt	NoCt	24.16	Consistent
49	Negative	Negative	NoCt	NoCt	NoCt	29.01	Consistent
50	Negative	Negative	NoCt	NoCt	NoCt	28.18	Consistent
51	Negative	Negative	NoCt	NoCt	NoCt	30.14	Consistent
52	Negative	Negative	NoCt	NoCt	NoCt	29.10	Consistent
53	Positive	Positive	30.77	29.76	29.99	26.16	Consistent
54	Positive	Positive	33.11	32.44	33.02	29.11	Consistent
55	Negative	Negative	NoCt	NoCt	NoCt	25.11	Consistent
56	Negative	Negative	NoCt	NoCt	NoCt	26.08	Consistent
57	Negative	Negative	NoCt	NoCt	NoCt	25.05	Consistent
58	Negative	Negative	NoCt	NoCt	NoCt	30.11	Consistent
59	Negative	Negative	NoCt	NoCt	NoCt	26.13	Consistent
60	Positive	Positive	29.32	28.66	29.54	28.01	Consistent
61	Positive	Positive	35.06	34.41	35.11	25.07	Consistent
62	Positive	Positive	27.30	26.22	26.56	28.16	Consistent
63	Positive	Positive	29.72	28.41	28.73	30.07	Consistent
64	Positive	Positive	24.67	23.11	24.98	28.11	Consistent
65	Positive	Positive	23.78	23.33	23.64	27.04	Consistent
66	Negative	Negative	NoCt	NoCt	NoCt	25.02	Consistent
67	Negative	Negative	NoCt	NoCt	NoCt	28.07	Consistent
68	Negative	Negative	NoCt	NoCt	NoCt	25.18	Consistent
69	Negative	Negative	NoCt	NoCt	NoCt	30.04	Consistent
70	Positive	Positive	31.02	30.20	30.58	24.00	Consistent
71	Positive	Positive	30.79	30.24	29.98	28.18	Consistent
72	Positive	Positive	27.33	26.55	26.76	29.17	Consistent
73	Negative	Negative	NoCt	NoCt	NoCt	24.00	Consistent
74	Negative	Negative	NoCt	NoCt	NoCt	27.10	Consistent
75	Negative	Negative	NoCt	NoCt	NoCt	25.05	Consistent
76	Positive	Positive	30.43	29.99	30.22	28.12	Consistent
77	Negative	Negative	NoCt	NoCt	NoCt	25.00	Consistent
78	Negative	Negative	NoCt	NoCt	NoCt	24.12	Consistent
79	Negative	Negative	NoCt	NoCt	NoCt	28.15	Consistent
80	Negative	Negative	NoCt	NoCt	NoCt	27.03	Consistent
81	Positive	Positive	29.35	28.68	28.58	25.06	Consistent
82	Positive	Positive	26.99	26.70	26.33	30.10	Consistent
83	Negative	Negative	NoCt	NoCt	NoCt	26.11	Consistent
84	Negative	Negative	NoCt	NoCt	NoCt	27.05	Consistent
85	Negative	Negative	NoCt	NoCt	NoCt	24.11	Consistent
86	Negative	Negative	NoCt	NoCt	NoCt	28.04	Consistent
87	Negative	Negative	NoCt	NoCt	NoCt	27.00	Consistent
88	Negative	Negative	NoCt	NoCt	NoCt	30.04	Consistent
89	Negative	Negative	NoCt	NoCt	NoCt	29.16	Consistent
90	Positive	Positive	32.89	32.22	32.47	24.02	Consistent
91	Negative	Negative	NoCt	NoCt	NoCt	26.01	Consistent
92	Negative	Negative	NoCt	NoCt	NoCt	29.00	Consistent
93	Negative	Negative	NoCt	NoCt	NoCt	29.08	Consistent
94	Negative	Negative	NoCt	NoCt	NoCt	25.16	Consistent
95	Negative	Negative	NoCt	NoCt	NoCt	28.01	Consistent
96	Negative	Negative	NoCt	NoCt	NoCt	26.00	Consistent
97	Negative	Negative	NoCt	NoCt	NoCt	24.17	Consistent
98	Negative	Negative	NoCt	NoCt	NoCt	24.03	Consistent
99	Negative	Negative	NoCt	NoCt	NoCt	27.13	Consistent
100	Negative	Negative	NoCt	NoCt	NoCt	26.03	Consistent

## Discussion

Since the twentieth century, the global outbreak of respiratory pathogens, such as influenza A virus and SARS-CoV-2, highlight the critical need for a fast and reliable diagnostic tool to identify infectious agents, which play a key role in controlling epidemics. Given its rapid and easy-to-use technique, RAT is applied largely in COVID-19 POCT as well as in the self-testing setting. However, the low sensitivity at disease onset hinders RAT from becoming an ideal disease-control tool^12,13^.

As nucleic acid amplification technology (NAAT) is analogous to PCR, isothermal amplification technology can amplify nucleic acids of the targeted pathogen with specific primers and has a theoretically higher sensitivity than rapid antigen test (RAT)^17,18^. Since the amplification process is at a constant temperature, the reaction time is much shorter than that of PCR. Combining these advantages, isothermal amplification becomes a promising candidate to develop a disease-control tool.

Current isothermal nucleic acid amplification techniques, such as nucleic acid sequence-based amplification (NASBA), exponential strand displacement amplification (E-SDA), exponential rolling circle amplification (E-RCA), LAMP, helicase-dependent amplification (HDA), recombinase polymerase amplification (RPA), and exponential amplification reactions (EXPAR)^19–24^, provide rapid and simple experimental procedures for nucleic acid-based diagnosis. Yet, these technologies still have several shortcomings. LAMP and EXPAR might have a risk of non-specific amplification, and it may take efforts to comprehensively eliminate false-positive amplicons caused by the accumulation of non-specific products^9^. The RPA system comprises a complex enzyme mix, and it is difficult to couple crude samples to the RPA system. RCA requires a circular template, often requires an additional ligation step, and its application is complicated. We examined the performance of RHAM and LAMP (Fig. 2), with RHAM demonstrating rapid and accurate detection of RNA samples, reflecting a promising platform for isothermal detection and POCT application.

In summary, we here demonstrate the development of a new isothermal amplification technology termed RHAM, which is based on RNase HII and LAMP, to detect targets rapidly and accurately. Further, with less complex sample preparation methods, RHAM can detect clinical samples as fast as 20 min, demonstrating its tremendous potential to develop a diagnostic tool in POC or even self-testing areas. However, further clinical samples should be analyzed to additionally demonstrate the sensitivity and specificity of the RHAM system.

## Materials and methods

### Virus samples

MERS coronavirus (ATCC, VR-3248SD), coronavirus OC43 (ATCC, VR-1558D), HKU1 (ATCC, VR-3262SD), 229E (ATCC, VR-740DQ), NL63 (ATCC, VR-3263SD), SARS coronavirus (ATCC, VR-3280SD), influenza A H1N1 (ATCC, VR-1469), H5N1 (ATCC, VR-1647), influenza B Virus (ATCC, VR-1735D), respiratory syncytial virus type A (ATCC, VR-1804DQ) and B (ATCC, 1850DQ), human parainfluenza virus type II (ATCC, VR-92DQ), adenovirus type 3 (ATCC, VR-3DQ) and 7 (ATCC, VR-7DQ), enterovirus EV71 (ATCC, VR-1775DQ), *Mycoplasma pneumoniae* (ATCC, 29342), human cytomegalovirus (ATCC, VR-1590), and *Mycobacterium tuberculosis* (ATCC 25177) were purchased from the ATCC SARS-CoV-2 RNA (GBW(E)091098) was purchased from the National Institute of Metrology (China, CNRN). SARS-CoV-2 clinical samples were collected by the KAVI-ICR laboratory in Kenya approved by the ethics committee.

### LAMP reaction

The LAMP primer set targeting the Orf1ab gene and N gene was designed by Primer Explorer (http://primerexplorer.jp/) and synthesized by Hippo Bio (Huzhou, China). The RHAM probe was designed using Primer 5.0 and synthesized by Hippo Bio (Huzhou, China). The LAMP reaction contained a final concentration of 1.6 μM FIP/BIP, 0.2 μM F3/B3, and 0.4 μM Loop F/B primers. 10 μmol of Syto9 fluorescent dye (ThermoFisher, S34854), 1× isothermal amplification buffer (20 mM Tris–HCl, 50 mM KCl, 10 mM (NH4)_2_SO_4_, 2 mM MgSO_4_ (Total 8 mM in Reaction), 0.1% Tween 20, pH 8.8), 1.4 mM dNTPs (New England Biolabs N0447), 8 U Bst DNA polymerase, large fragment [Bst DNA Polymerase, Large Fragment (New England Biolabs M0275)], 7.5 U WarmStart RTx Reverse Transcriptase (New England Biolabs M0380), and 10 μL templates or samples were included in a 25 μL reaction. The LAMP reaction was performed at 65 °C on a qPCR machine (SLAN-96P^®^, Hongshi, Shang Hai Hongshi Medical Technology Co., Ltd, Shanghai, China) for 40 min, with fluorescence measurements every 60 s. Primers: Orf1ab-F3: 5′-ACTTGTTTCAAC ATGCCAAT-3′; Orf1ab-B3: 5′-GTAGCTTGTTTACCACACG-3′; Orf1ab-FIP: 5′-AGGGTTGTCTGCTGTTGTCCTTAGATTCTTGCAAAAGAGTCT-3′; Orf1ab-BIP: 5′-AGGGTGTAGAAGCTGTTATGTACATACAAGGTATCTGAACACCTTT C-3′; Orf1ab-LF: 5′-ACAAGTTTTACACACCACGTTC-3′; Orf1ab-LB: 5′-TGGG CACACTTTCTTATGAAC-3′; N gene-F3: 5′-CCAGAATGGAGAACGCAGTG-3′; N gene-B3: 5′-CCGTCACCACCACGAATT-3′; N gene-FIP: 5′-AGCGGTGAACC AAGACGCAGGGCGCGATCAAAACAACG-3′; N gene-BIP: 5′-AATTCCCTCG AGGACAAGGCGAGCTCTTCGGTAGTAGCCAA-3′; N gene-LF: 5′-TTATTGGG TAAACCTTGGGGC-3′; N gene-LB: 5′-TAACACCAATAGCAGTCCAGATGA-3′.

### RHAM reaction

RNase HII reporting system signal was verified by adding 5 U RNase HII nuclease (New England Biolabs, M0288), 5 pmol probe (6-FAM-dT or 6-FAM-dC is used as the quenching group and 3′-BHQ1 or BHQ1-dT as the fluorophore.), and 0.5 pmol complementary chain template to 25 μL of LAMP reaction system, reacting on the SLAN-96P^®^ platform at 65 °C for 40 min, with fluorescence measurements performed every 60 s. For RHAM detection, 5 U RNase HII and 5 pmol probes were included with 1.6 μM FIP/BIP, 0.2 μM F3/B3, and 0.4 μM Loop F/B primers. 10 μmol of Syto9 fluorescent dye (ThermoFisher, S34854), 1 × isothermal amplification buffer (20 mM Tris–HCl, 50 mM KCl, 10 mM (NH_4_)_2_SO_4_, 2 mM MgSO_4_ (Total 8 mM in Reaction), 0.1% Tween 20, pH 8.8), 1.4 mM dNTPs (New England Biolabs N0447), 8 U Bst DNA polymerase, large fragment [Bst DNA Polymerase, Large Fragment (New England Biolabs M0275)], 7.5 U WarmStart RTx Reverse Transcriptase (New England Biolabs M0380), and 10 μL templates or samples to make a 25 μL one-pot RHAM reaction. The RHAM reaction was performed at 65 °C on the SLAN-96P^®^ platform for 40 min, with fluorescence measurements performed every 60 s. Orf1ab-Probe: 5′-GGGCACACTTTCTTA/i6FAMdT/GA/rA/CAATTTA-3′-BHQ1; N gene-Probe: 5′-AGGTCTTCC/iBHQ1dT/T/rG/C/i6FAMdC/ATGTTGAGT-3′.

### SARS-CoV-2 clinical sample collection and testing

Nasopharyngeal swabs were collected and transported using a virus sampling tube with transport medium (MT0301-2, YOCON Biotechnology Co., Ltd.), which was further submitted to the KAVI-ICR laboratory in Kenya. The RNA was processed by an RNA extraction kit (TIANamp Virus DNA/RNA Kit, DP315, TIANGEN BIOTECH (BEIJING) CO., LTD, Beijing, China), sample lysis buffer (Tris–HCl pH 8.8 10 mM, NP-40 0.5% (v/v), Tween-20 0.15% (v/v), Antifoam SE-15 0.01% (v/v). SARS-CoV-2 was determined using the Novel Coronavirus 2019-nCoV Nucleic Acid Detection Kit (fluorescent PCR) (Liferiver, Shanghai, China), which contained primers and probes for the novel coronavirus (2019-nCoV) Orf1ab, N, and E genes. The three viral targets were qualitatively detected on the SLAN96P^®^ platform. The KAVI Clinical Research Institute in Kenya was commissioned to use SARS-CoV-2 clinical samples to evaluate the performance of the RHAM system on the SLAN-96P^®^ platform.

### Ethical declarations

All experimental protocols and clinical samples were approved the ethics committee of KAVI Clinical Research Institute in Kenya. All methods were carried out in accordance with relevant guidelines and regulations. We received written informed consent of all participants.

### Supplementary Information

Supplementary Figure 1.

## Data Availability

The data that support the findings of this study are available from the corresponding authors upon reasonable request.

## References

[1] Crum RJ (2023). Mitigation of influenza-mediated inflammation by immunomodulatory matrix-bound nanovesicles. Sci. Adv..

[2] Islam A (2023). Epidemiology and molecular characterization of avian influenza A viruses H5N1 and H3N8 subtypes in poultry farms and live bird markets in Bangladesh. Sci. Rep..

[3] Benoit P (2021). Impact of cobas PCR Media freezing on SARS-CoV-2 viral RNA integrity and whole genome sequencing analyses. Diagn. Microbiol. Infect. Dis..

[4] Nyaruaba R (2022). Digital PCR applications in the SARS-CoV-2/COVID-19 era: A roadmap for future outbreaks. Clin. Microbiol. Rev..

[5] Deepak S (2007). Real-time PCR: Revolutionizing detection and expression analysis of genes. Curr. Genom..

[6] Listed N (1993). Prenatal interphase fluorescence in situ hybridization (FISH) policy statement. Am. J. Hum. Genet..

[7] Richards S (2015). Standards and guidelines for the interpretation of sequence variants: A joint consensus recommendation of the American College of Medical Genetics and Genomics and the Association for Molecular Pathology. Genet. Med..

[8] Wilgenbus KK, Lichter P (1999). DNA chip technology ante portas. J. Mol. Med. (Berl).

[9] He S (2022). Isothermal amplification based on specific signal extraction and output for fluorescence and colorimetric detection of nucleic acids. Talanta.

[10] Zhao Y, Chen F, Li Q, Wang L, Fan C (2015). Isothermal amplification of nucleic acids. Chem. Rev..

[11] Craw P, Balachandran W (2012). Isothermal nucleic acid amplification technologies for point-of-care diagnostics: A critical review. Lab Chip.

[12] Heydecke A, Gullsby K (2023). Evaluation of the performance of a rapid antigen test (Roche) for COVID-19 diagnosis in an emergency setting in Sweden. J. Med. Virol..

[13] Xie JW (2023). Nasal swab is a good alternative sample for detecting SARS-CoV-2 with rapid antigen test: A meta-analysis. Travel Med. Infect. Dis..

[14] Dobosy JR (2011). RNase H-dependent PCR (rhPCR): Improved specificity and single nucleotide polymorphism detection using blocked cleavable primers. BMC Biotechnol..

[15] Van Ness J, Van Ness LK, Galas DJ (2003). Isothermal reactions for the amplification of oligonucleotides. Proc. Natl. Acad. Sci. USA.

[16] Notomi T, Mori Y, Tomita N, Kanda H (2015). Loop-mediated isothermal amplification (LAMP): Principle, features, and future prospects. J. Microbiol..

[17] Dong K (2021). A loop-mediated isothermal amplification with a nanoparticle-based lateral flow biosensor assay to detect pseudomonas aeruginosa in endophthalmitis. Transl. Vis. Sci. Technol..

[18] Sadeghi Y (2021). The sensitivity and specificity of loop-mediated isothermal amplification and PCR methods in detection of foodborne microorganisms: A systematic review and meta-analysis. Iran. J. Public Health.

[19] Malek L, Sooknanan R, Compton J (1994). Nucleic acid sequence-based amplification (NASBA). Methods Mol. Biol..

[20] Walker GT, Little MC, Nadeau JG, Shank DD (1992). Isothermal in vitro amplification of DNA by a restriction enzyme/DNA polymerase system. Proc. Natl. Acad. Sci. USA.

[21] Daubendiek SL, Ryan K, Kool ET (1995). Rolling-circle RNA synthesis: Circular oligonucleotides as efficient substrates for T7 RNA polymerase. J. Am. Chem. Soc..

[22] Notomi T (2000). Loop-mediated isothermal amplification of DNA. Nucleic Acids Res..

[23] Vincent M, Xu Y, Kong H (2004). Helicase-dependent isothermal DNA amplification. EMBO Rep..

[24] Piepenburg O, Williams CH, Stemple DL, Armes NA (2006). DNA detection using recombination proteins. PLoS Biol..

