# Supplementary material for: Rapid and accurate detection of SARS-CoV-2 using the RHAM technology
Source: Sci Rep. 2023 Dec 20;13:22798. doi: 10.1038/s41598-023-49733-7 (PMC10739982; doi:10.1038/s41598-023-49733-7)
Supplement: Supplementary file 1 — Supplementary Figure 1. [file 41598_2023_49733_MOESM1_ESM.pptx]

## Slide 1
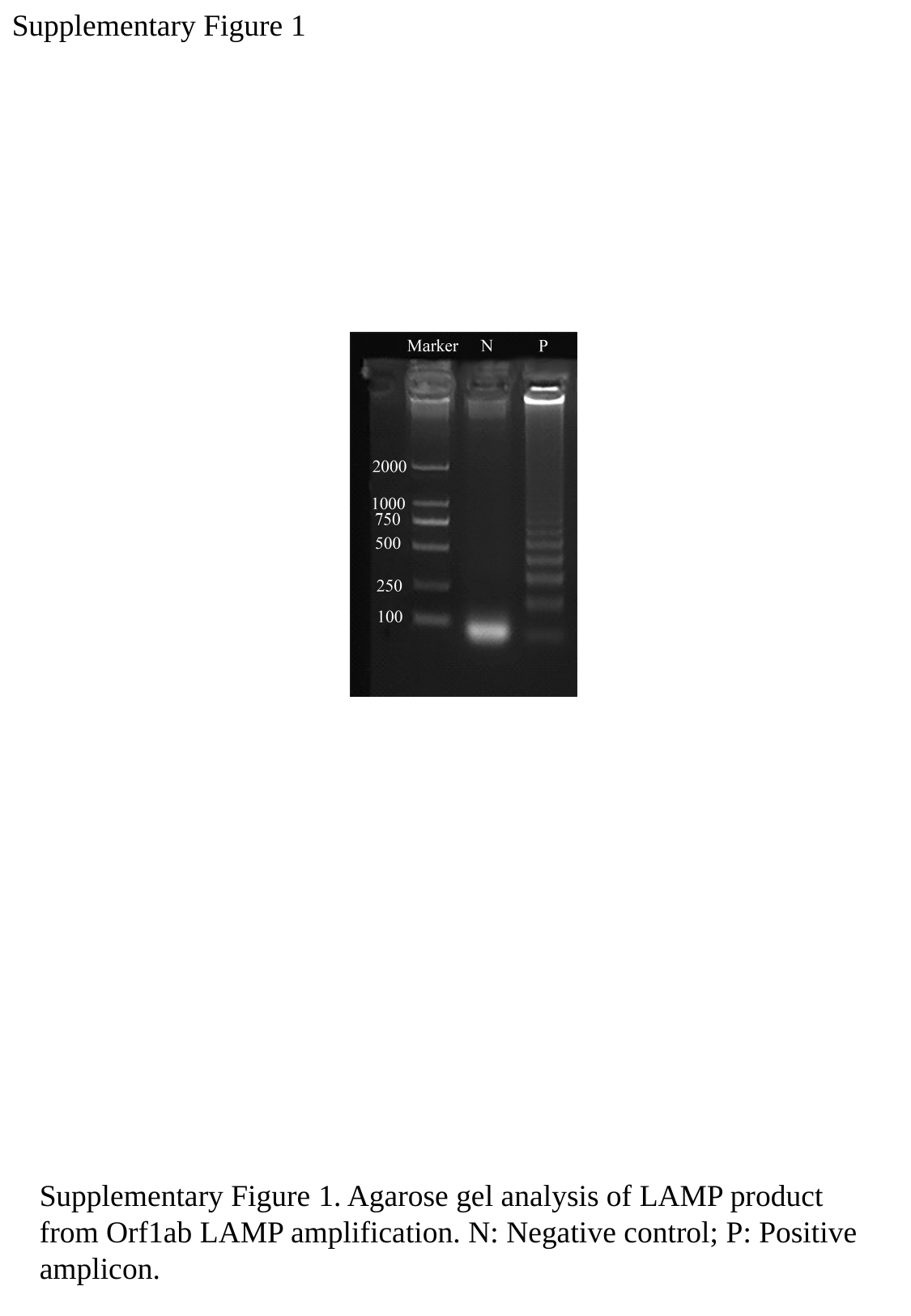

Supplementary Figure 1
Supplementary Figure 1. Agarose gel analysis of LAMP product from Orf1ab LAMP amplification. N: Negative control; P: Positive amplicon.
